# Comparing Multiple Criteria for Species Identification in Two Recently Diverged Seabirds

**DOI:** 10.1371/journal.pone.0115650

**Published:** 2014-12-26

**Authors:** Teresa Militão, Elena Gómez-Díaz, Antigoni Kaliontzopoulou, Jacob González-Solís

**Affiliations:** 1 Institut de Recerca de la Biodiversitat (IRBio) i Departament de Biologia Animal, Universitat de Barcelona, Barcelona, Spain; 2 Department of Biology, Emory University, Atlanta, Georgia, United States of America; 3 CIBIO Research Centre in Biodiversity and Genetic Resources, InBIO, Universidade do Porto, Campus Agrário de Vairão, Vairão, Vila do Conde, Portugal; 4 Department of Ecology, Evolution, and Organismal Biology, Iowa State University, Ames, Iowa, United States of America; University of Colorado, United States of America

## Abstract

Correct species identification is a crucial issue in systematics with key implications for prioritising conservation effort. However, it can be particularly challenging in recently diverged species due to their strong similarity and relatedness. In such cases, species identification requires multiple and integrative approaches. In this study we used multiple criteria, namely plumage colouration, biometric measurements, geometric morphometrics, stable isotopes analysis (SIA) and genetics (mtDNA), to identify the species of 107 bycatch birds from two closely related seabird species, the Balearic (*Puffinus mauretanicus*) and Yelkouan (*P. yelkouan*) shearwaters. Biometric measurements, stable isotopes and genetic data produced two stable clusters of bycatch birds matching the two study species, as indicated by reference birds of known origin. Geometric morphometrics was excluded as a species identification criterion since the two clusters were not stable. The combination of plumage colouration, linear biometrics, stable isotope and genetic criteria was crucial to infer the species of 103 of the bycatch specimens. In the present study, particularly SIA emerged as a powerful criterion for species identification, but temporal stability of the isotopic values is critical for this purpose. Indeed, we found some variability in stable isotope values over the years within each species, but species differences explained most of the variance in the isotopic data. Yet this result pinpoints the importance of examining sources of variability in the isotopic data in a case-by-case basis prior to the cross-application of the SIA approach to other species. Our findings illustrate how the integration of several methodological approaches can help to correctly identify individuals from recently diverged species, as each criterion measures different biological phenomena and species divergence is not expressed simultaneously in all biological traits.

## Introduction

Species have long been recognized as one of the fundamental units of biology. After endless debates and controversies about the concept of species, the emphasis is now shifting towards species delimitation [1]–[3]. Different species characteristics are acquired at different times during the process of lineage divergence and once two evolutionarily distinct lineages became phenotypically distinguishable, diagnosable, reciprocally monophyletic, reproductively incompatible, ecologically distinct, it is generally accepted that there are two species [4]. However, recently diverged species may not yet have acquired the entire set of these characteristics and as a consequence are often difficult to tease apart. While discussion of different operational methods to identify species has previously been restricted mostly to taxonomists, species delimitation and identification is now underpinned by a growing concern over threats to biodiversity [2], [5]. Although some national bodies allow the use of taxonomical entities below the species level (e.g. [6]), species is the key unit for conservation biology since it is the basic unit used in international conventions and agreements and for providing lists of threatened organisms [7]. Since many human activities are responsible for the direct death of billions of animals each year [8], the unequivocal identification of individuals to the species level is crucial for an accurate evaluation of the anthropogenic impact, particularly among threatened species.

Despite the important taxonomic and conservation implications of correct species delimitation and identification, there is no universal consensus on how this task should be accomplished, although numerous approaches have been proposed [1], [9]. Traditional tools to identify species, such as morphology and colouration, typically rely on the comparison of phenotypic traits of specimens of unknown identity to specimens of reference of known species [10]. Such comparative approaches pose several limitations to identify recently diverged species that show little phenotypic differentiation, and the decision on the criteria to use is often subjective and possibly controversial [11]. Recently, geometric morphometric analysis, which aims to characterize form and quantify morphological variation in scrupulous detail, has proven useful for fine-scale studies of species discrimination [12], [13]. This approach allows a formal mathematical definition of shape and size, while facilitating the study of shape variation in an objective way. However, the use of shape traits in taxonomy and systematics has also been questioned [14], [15], since organismal shape is quite plastic and can be easily subject to variation due to many factors other than specific identity (i.e. environmental factors, ontogenetic effects).

A widely used alternative to morphological traits for systematic inference and species discrimination has been the implementation of neutral genetic markers. DNA sequencing has emerged as a powerful approach for species definition and identification and for biodiversity assessment [16]. This method uses a short genetic marker to identify it as belonging to a particular species [17]. Mitochondrial DNA (mtDNA) is the marker of choice in most studies because it occurs in a high number of copies in cells and it has rapid mutation rates and shorter coalescent times compared to nuclear DNA [18]. Moreover, compared to other genetic markers, mtDNA is relatively easy, rapid, and inexpensive to sequence. However, it also has some drawbacks. Its maternal inheritance and the fact that it is a single-inheritance unit, can lead to introgression and incomplete lineage sorting [18], as well as incongruence between mitochondrial and nuclear genes in inferring species relationships [9], [19], [20]. These limitations are especially problematic in the identification of recently diverged and hybridizing species.

Another way to identify species is to use ecological or behavioural criteria based on different aspects such as bioacoustics, reproductive strategies, or migration patterns. Stable isotope analysis (from now on referred to as "SIA") can be used as an ecological criterion and it has been shown to be a powerful tool to investigate feeding habits and migratory movements in a number of animal taxa [21]. As a species discrimination tool, SIA can be useful if different species have different diets, or feed in areas with distinct isotopic baselines [21], even if such species share habitat, or show similarity in other evolutionarily or ecologically relevant traits (i.e. morphology and genetics; [22], [23]). However, stable isotope values of predators can be affected by extrinsic factors such as temporal variation at the baseline level [24]. Therefore the suitability of SIA for species identification and taxonomical purposes needs to be thoroughly explored case-by-case.

Given that no single method of species identification has proven entirely successful, nowadays many studies defend the use of the integrative taxonomy, which consists in combining multiple disciplines, such as morphometrics, genetics, ecology and behaviour, to identify and delimit species [1], [25], [26]. A case example of a pair of sibling species difficult to tease apart occurs within the Procellariiformes: the Yelkouan (*Puffinus yelkouan* (Acerbi 1982), from now on referred to as "YS") and Balearic (*P. mauretanicus* Lowe 1921, from now on referred to as "BS") shearwaters. Despite the fact that they are now considered different species [27]–[33], the two taxa overlap in a number of phenotypic, behavioural and genetic traits [34]. They exhibit a noticeable overlap in plumage colouration [35] and body size [36], [37]. Moreover, recent genetic analyses on these species reported 2.2–2.9% of inter-specific divergence [33] (which is a slight smaller value than other well-recognized seabird taxa [33]), as well as, evidence of maternal introgression and potential cases of natural hybridization in contact areas [38]. The only aspect in which the two species have minimal overlap is in their non-breeding areas. In general, BS migrates to the Atlantic after breeding [39], whereas YS migrates to the Black and Aegean Seas [40], [41]. However an important number of YS do not migrate but rather remain near the breeding colonies year-round [40]–[42]. Isotopic data for BS also suggests that a few individuals may not migrate and so it is possible a certain degree of overlap between the two species in the Western Mediterranean Sea [42].

**Figure 1:**
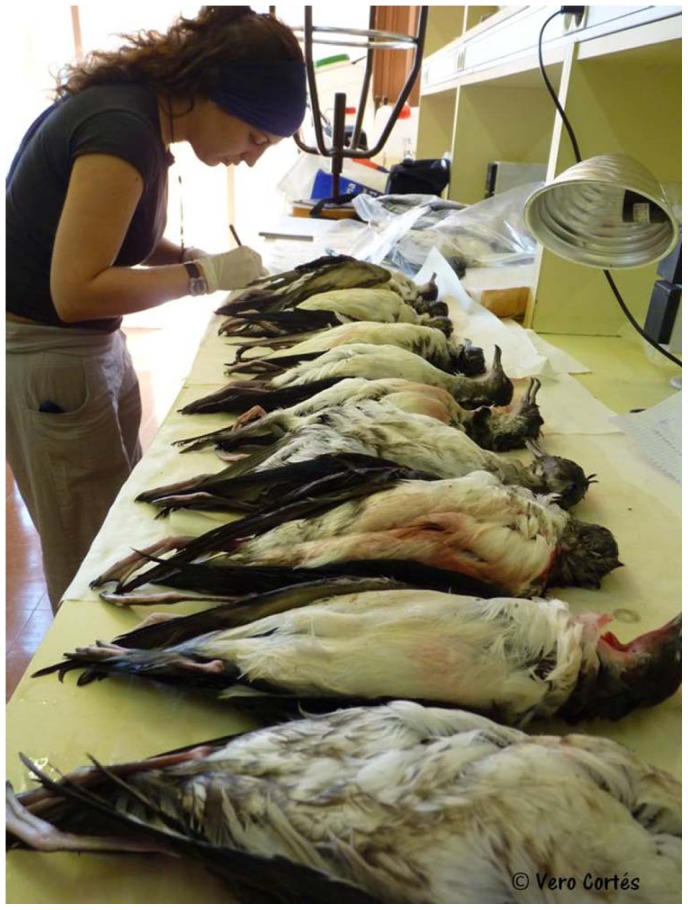
Exemplars of a massive bycatch of Yelkouan and Balearic shearwaters by longliners in Catalonia coast. Some exemplars of a massive bycatch of Yelkouan and Balearic shearwaters by longliners in Catalonia coast, where it is possible to see their wet and bloody plumage. Picture courtesy of Verónica Cortés.

Finally, the correct identification of these two species is of particular conservation concern, since BS and YS are catalogued, respectively, as critically endangered and vulnerable [43], and are bycaught in large numbers by longline fisheries (Fig. 1, [44], [45]). Thus, there is an urgent need to estimate bycatch rates of each species accurately as they may be differentially affected by longliners. However, the difficulties in identifying the species of all dead specimens only by plumage coloration or biometric measurements preclude a detailed assessment of the impact of this fishery practice on each species.

**Figure 2:**
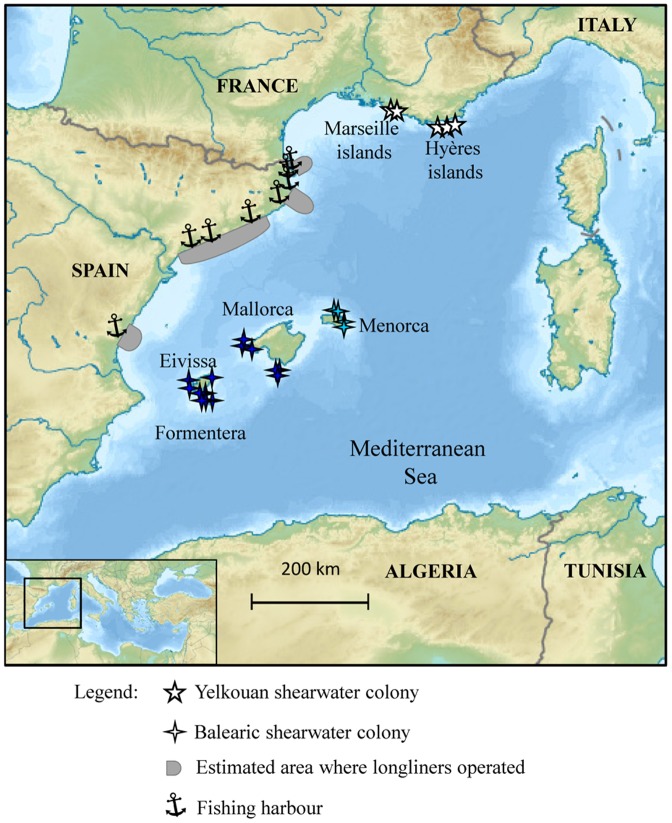
Map with the location of bycatch birds and the breeding colonies of the Balearic and the Yelkouan shearwaters in the Western Mediterranean. The grey areas represent the areas where longliners from the NE coast of Spain operate and in which bycatch birds were accidentally caught. These areas overlap with the foraging grounds of Balearic shearwaters from Balearic Islands (Eivissa, Formentera, Mallorca and Menorca, [39], [88], [89]) and with those of Yelkouan shearwaters from Marseille and Hyères islands (France, [40]). There are more colonies of Yelkouan shearwaters in the Western Mediterranean but present evidences suggest they do not forage in the NE coast of Spain [90].

The main aim of this study was to evaluate the utility and consistency of plumage colouration, morphological, isotopic and genetic traits for distinguishing individuals from these recently diverged species. For this purpose, we examined 107 BS and YS specimens accidentally caught by longline fisheries in the NE coast of Spain (Western Mediterranean, see Fig. 2). On this dataset, we applied a comparative approach to assess the suitability and mismatch of species identification in all birds by all criteria, and using birds of known origin as a reference for species identity, whenever possible.

## Materials And Methods

We examined plumage colouration, linear biometric measurements, geometric morphometrics of bill shape, mitochondrial DNA control region sequences, and carbon and nitrogen stable isotopes from feathers of 107 specimens believed to be either Yelkouan (YS) or Balearic (BS) shearwaters. These birds were accidentally caught in the period from 2003–2008 by local longline fisheries operating in the NE coast of Spain (see Fig. 2; a subsample of these birds was already used to infer the non-breeding areas of both species, [42]). All birds were maintained frozen at −20°C until dissection. During dissection, we determined their sex and collected muscle or liver tissue samples for genetic and feathers for isotopic analyses, as well as photographs for geometric morphometric analysis. Moreover, in this study we only included birds without bursa of Fabricius (which is only present in juvenile and immature, [46]), to avoid the potential confounding effects of juvenile/immature birds in biometry and stable isotope values.

Additionally, we included 10 YS sampled in 2006 at their breeding grounds in Hyères Islands (see Fig. 2; Porquerolles and Port-Cros, France) as reference specimens of known origin and species, for biometric, genetic and SIA. For BS one of the bycaught birds was also used as reference specimens for all criteria as this bird was ringed in a BS colony (Mallorca Island, see more details in results). Moreover, exclusively for genetic identification we also used another YS from Hyères Islands sampled in 2005 and reference specimens of BS from GenBank (see details below).

Finally, we assessed the plumage colouration, biometric measurements and SIA in 77 additional bycatch shearwaters (see dataset 2 Table 1) bycaught in the following years. With these additional birds, we spanned the temporal series of bycaught specimens that moulted their feathers from 2002 to 2011 (see Table 1). All birds from both datasets for which it was possible to infer their species (see more details below about species identification) were then used to check the temporal stability in stable isotope values (see for e.g. [47]).

**Table 1:** Number of Yelkouan (YS), Balearic (BS)and unknown shearwaters that moulted their feathers in the same year.

Year that birds moulted their feathers	Bycatch Yelkouan shearwaters (YS)	Bycatch Balearic shearwaters (BS)	Bycatch unknown shearwaters	Yelkouan shearwaters from Hyères (France)
Dataset 1				
2002	1	3	0	0
2003	0	0	0	0
2004	0	4	1	0
2005	16	0	1	10
2006	24	17	1	0
2007	0	38	1	0
Dataset 2				
2008	0	5	1	0
2009	4	5	2	0
2010	17	21	8	0
2011	0	13	1	0

Bird species from dataset 1 was inferred based in the agreement in species assignment of at least three of the following criteria: plumage colouration, biometric measurements, genetic and stable isotope data (see table 2). Birds with less than 3 criteria agreeing in species assignment were considered unknown shearwaters. Bird species from dataset 2 was inferred based on the agreement on species assignment by plumage colouration, discriminant functions of biometric measures and stable isotope data. Birds from dataset 2 were considered unknown shearwaters if one of the criteria disagreed in species identification. All birds from dataset 1 were used in cluster analysis (except the 10 Yelkouan shearwaters (YS) from Hyères Islands (France), which were not used in geometric morphometric cluster analysis). All birds from dataset 1, expect unknown shearwaters, were used in species identification and discriminant function. All birds from both dataset, except the unknown shearwaters, were used for checking annual variation in stable isotope data.

### Plumage colouration

In birds, plumage colouration is the most common tool for species identification and therefore we included this criterion for species assignment following current descriptions of each species [35], [48]. We carefully checked the colour of the underparts, which is pure white in the YS but ranges from white to completely brown in the BS. We also examined the colour boundary between the under and upperparts, which is well marked in the YS but diffuse in the BS, especially around the neck. However, the wet and bloody plumage (see Fig. 1) of the bycaught birds precluded a reliable quantitative evaluation of the plumage colouration. Moreover, about 10% of BS are known to have white underparts similar to the YS [35], being hard to distinguish from the latter species and some bycaught birds were difficult to assign to one of the species. These birds are referred to from now on as “doubtful plumage” birds (see results).

### Biometric and geometric morphometric analyses

To quantify global morphological variation for each individual, we measured six biometric variables typically used for shearwater species identification: bill depth at base, bill depth at nostril, bill length, maximum head length, right tarsus length and right wing length compressed (for more detailed information, see S1 Fig.). All measurements were taken using a digital calliper (±0.01 mm), except for wing length, which was measured using a ruler (±0.5 mm; for biometric measurements of each individual see S1 Table). In order to quantify and control for measurement error, both random and systematic (e.g. due to various observers), replicate measurements were taken twice on a subset of birds (sample size from 37 to 85 birds, see S2 Table) by two different observers (TM and JGS). The intra- and inter-observer errors were evaluated using intra-class correlations. Although consistency between replicates of the same observer and across observers was always higher than 0.9 (from 0 to 1, S2 Table), there were significant differences between measurements and observers (for paired-sampled t test results see S2 Table). For all subsequent analysis, we thus chose to use the first measurement of observer 1 (TM).

The bill is the only external anatomic structure rigid enough to be used for locating landmarks unambiguously (i.e., specific points on a biological structure) and capture its shape in both alive and dead birds, potentially allowing its use in field studies. We therefore chose this structure to perform geometric morphometric analyses on bycatch birds of dataset 1. To capture bill shape, we took high-resolution photographs of the right side of the bill using a digital camera (Nikon D200 AF-S Micro Nikkor 60mm f/2.8GED Nikon), mounted on a tripod and adjusted to ensure the objective was always parallel to the bill's lateral surface. Graph paper was placed underneath the bill to record scale.

**Figure 3:**
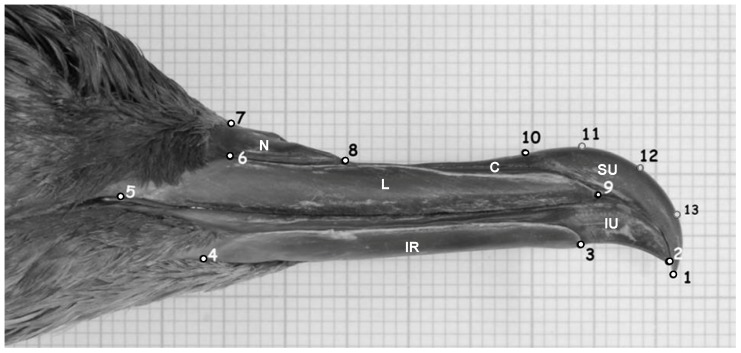
Location of the landmarks (1–10) and semilandmarks (11–13) used to delineate bill shape. N: nares, C: culminicorn, L: latericorn, SU: superior unguicorn, IU: inferior unguicorn, IR: inferior ramicorn (see [91] and S3 Table).

Some biological structures, such as the tip of the bill, often have smooth parts which cannot be characterized only by landmarks but can still give us important information. A method of capturing the information of these rounded parts is through points on the outline contour, the semilandmarks, which are points free to slide along an outline [49], [50]. So to record bill shape we used tpsDig software [51] to digitize 10 landmarks and three equidistant semilandmarks (11, 12 and 13 in Fig. 3 and see S3 Table), located between landmarks 1 and 10 along the outline contour of the superior unguicorn. To avoid any bias in the procedure, all bill photographs, as well as the landmark digitizing, were always done by the same person (TM).

Landmark configurations were then superimposed in tpsRelW [52] using a Generalized Procrustes Analysis (GPA; [53]) in order to remove size, translate, rotate the landmark coordinates and slide the semilandmarks (11, 12 and 13) along the curve of the superior unguicorn. Semilandmarks were slided using the minimum Procrustes distance criterion [54]. To obtain shape variables while accounting for the degrees of freedom lost due to the use of semilandmarks, we performed a relative warps analysis in tpsRelw [52]. We used the resulting relative warps with non-zero eigenvalues (19 in total) as shape variables for subsequent analyses. To represent size, we used centroid size (CS), which is the square root of the summed squared distances of each landmark from the centroid of the landmark configuration. CS is a measure of size mathematically independent of shape and is uncorrelated to it in the absence of allometry [55].

### Genetic analyses

DNA was isolated from liver or muscle tissues. A 293-bp fragment of the hypervariable Domain I of the mitochondrial control region of *Puffinus* was amplified and sequenced (see [56]) for primers, amplification conditions and laboratory protocols). Negative and positive controls were included in each of the amplification runs. Extraction, amplification and sequencing replicates were performed as quality assurance and control. Chromatograms were checked manually, assembled and edited using Geneious v. 5.3.6 (Biomatters Ltd.). Sequences were aligned for each gene independently using the online version of MAFFT v.6. (http://mafft.cbrc.jp/alignment/server/index.html) with default parameters (gap opening penalty  = 1.53, gap extension  = 0.0). In addition to analytical controls, and to assess the reliability of the data, the obtained sequences were compared with previously published data on the studied species and all variable sites were confirmed by visual inspections of the chromatograms.

### Stable isotope analyses (SIA)

To interpret the isotopic values of feathers it is essential to know when and where the feathers have been moulted. In general, the renewal of flight feathers in the BS occurs from May to October, lasts for about three months and starts about one month earlier than in the YS [57]. However some birds, such as immature, may moult earlier than adult birds, therefore only adult birds were included in this study. To differentiate the YS and the BS using SIA, we used two feathers: one that grew at the beginning of the non-breeding period (the 1^st^ primary, P1; author's personal observation) and another that in shearwaters tends to grow at the end (6^th^ rectrix, R6). Since the majority of birds of these species differ in their migration areas during the non-breeding period [39]–[41], where they moult most of their flight feathers [42], we expect that the two species will show different isotopic values. We thus analyzed the stable isotopes of carbon (*δ* ^13^C) and nitrogen (*δ* ^15^N) from the entire feathers P1 and R6. Preparation and analyses of feathers were carried out following [58] and the "principle of identical treatment" [59]. Isotope ratios are expressed conventionally as *δ* values in part per thousand (‰) according to the following equation:
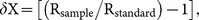
where X (‰) is ^13^C or ^15^N and R are the corresponding ratio ^13^C/^12^C or ^15^N/^14^N related to the standard values. R_standard_ for ^13^C is Vienna Pee Dee Belemnite (VPDB) and for ^15^N is atmospheric nitrogen (AIR). The isotopic ratio mass spectrometry facility at the Serveis Científico-Tècnics of the University of Barcelona (Spain) applied international standards (IAEA CH_7_, IAEA CH_6_ and USGS 40 for C and IAEA N1, IAEA N2, IAEA NO_3_ and USGS 40 for N) and inserted two standard material samples every 12 feather samples to calibrate the system and compensate for any drift over time. Replicate assays of standard material samples indicated standard deviation of ±0.2 for carbon and nitrogen (see S4 Table).

### Cluster analyses

All cluster analysis of biometric, geometrics morphometric, SIA and genetic data were performed using R 3.1.0 [60]. For these cluster analyses, we used all birds from dataset 1, except in geometric morphometric for which was not possible to include the 10 YS from Hyères Islands (France). Before cluster analysis, to account for the effect of sexual size dimorphism, biometric measurements of males and females were standardized using the translation method which consists in subtracting the mean of each sex for all biometric traits [61]. Moreover to genetic data, we used the function "dist. alignment" from the package "seqinr" [62] to compute a matrix of pairwise distances from the aligned mitochondrial sequences (see above). The resulting matrix contains the squared root of the pairwise distances. For the cluster analysis of the genetic data, we included reference sequences for the mitochondrial control region of BS and YS of known origin (GenBank Accession No. DQ230131- DQ230217 and No. KP235423, KP235461, KP235462, KP235477-KP235483, respectively; [38], this study, see S5 Table), that we compared with sequences of bycatch individuals analyzed in this study (GenBank Accession No. KP235367-KP235371, KP235373-KP235395, KP235397-KP235404, KP235406, KP235407, KP235421, KP235423, KP235443-KP235461, KP235463-KP235476).

We estimated the optimal number of clusters for each quantitative criterion (biometric measurements, geometric morphometrics, stable isotopes and genetic data) based on the optimum average silhouette width using partitioning around medoids clustering method (function "pamk" from package "fpc", [63]). Subsequently, we performed the k-means cluster analysis based on the number of clusters previously estimated and using the function "clusterboot" (runs  = 10000, k = 2 and cluster method  =  pamkCBI), also included in the package "fpc" [63]. With this function, we also assessed the cluster stability based on the Jaccard's similarity index by using bootstraping resampling scheme. Generally, a valid, stable cluster should generate a mean Jaccard's similarity value of 0.75 or greater [63].

The results of each cluster analysis were plotted using the coordinates obtained from multidimensional scaling analysis ("cmdscale" function in the R base package, [64]) computed based on the Euclidean distance among birds. For genetic data, a minimal additive constant c* was computed such that the dissimilarities were Euclidean [65].

### Differences between species

To test differences between species we used all birds from dataset 1, except the ones that were not possible to infer their species. Linear biometric measurements and shape variables were normally distributed and variances did not differ among species and variables (Kolmogorov-Smirnov normality and Levene's test, respectively, p>0.05 in all cases). To test for differences in biometry and in geometric morphometric between species and sexes, all biometric variables and all relative wraps, respectively, were analyzed using a MANOVA design with specie and sex as fixed factors.

Regarding the centroid size data, it was logarithmically transformed and analyzed using a two-way ANOVA with species and sex as fixed factors.

Nitrogen isotopic data were normally distributed but carbon data showed a slightly bimodal distribution most likely because birds migrated to different areas with prey with distinct carbon values [66]–[70]. Since it was not possible to determine non-breeding areas of dead birds (independently from its stable isotope values) and departure from normality in carbon values was mild, we checked for differences in isotopic variables and feathers between species using pair-wise Student t-test. For variables that did not show homoscedasticity, we used the t-test results for unequal variances assumed.

### Annual variation in stable isotope values

To check the existence of annual isotopic variation on the feathers of the study species, we performed additional analysis to increase the temporal series of birds with stable isotope data. For this, we assessed the plumage colouration, biometric measurements and SIA in 77 additional bycatch shearwaters (dataset 2) that were bycaught in the following years of birds of dataset 1. However to infer the species of these additional shearwaters, we first performed a discriminant function analysis (DFA) based on biometric measures and stable isotope data (separately) of all birds (excluding the unknown shearwaters) of dataset 1. We carried out the DFA using 80% of the birds of each species (40 YS and 50 BS) to perform the DFA (training data) and the remaining 20% of the birds (11YS and 12BS) were used to test the efficiency of the DFA obtained (test-data). To construct the DFA we assumed equal prior probabilities and used the jacknife cross-validation. Next, we applied the obtained DFA of stable isotopes and biometric measures to the birds of the dataset 2 to infer their species based on the agreement between both DFA and plumage colouration.

Finally, to understand how annual variation in stable isotope values could bias species identification, we used all birds (excluding the unknown shearwaters) from the dataset 1 and those from the dataset 2 that were assigned to the same species by plumage colouration and DFAs of biometric measures and stable isotopic data. Then, we assigned each bird to the year when feathers were moulted rather than the year of capture (see Table 1), as stable isotope are integrated during the formation of the feather. The study species moult from May to October [57] and therefore feathers collected from March to June were assigned to the previous year, whereas feathers collected from October to November were assigned to the year of collection. Only birds that were not moulting primary or the R6 feathers were included in this study. We then performed a restricted maximum likelihood estimation of variance components (VARCOMP) to understand the contribution of each random effect, here species and year of moult, to the variance of the dependent variable, i.e. carbon and nitrogen isotopic values of each feather. Moreover, we also represented the isotopic data separated by species, feather and year of moult (only for years with samples size greater than 10 birds).

All statistical procedures were performed using PASW 18 [71] except the cluster analysis which were performed using R 3.1.0 [60]. For all tests we assumed a critical P value of 0.05, except for the tests in which we applied the Bonferroni correction.

### Ethics statements

All animals were handled in strict accordance with good animal practice as defined by the current European legislation, and all animal work was approved by the respective regional committees for scientific capture (prefecture of the Var region (authorisation n°7/2004) and CRBPO (National Museum of Natural History, Paris)). The person present in Fig. 1 of this manuscript has given written informed consent (as outlined in PLOS consent form) to publish these case details.

## Results

Among the 107 collected bycatch specimens of the dataset 1, two birds carried a ring and both were ringed in the Balearic Islands (see S6 Table). Bird number 5042990 was ringed on the 01/07/1997 at the Sa Cella colony (Andratx, Mallorca) as a chick, so this bird can be reliably assigned as BS. Bird number 5070034 was ringed in Menorca, the only known colony where the two species are known to hybridize and the majority of birds are phenotypically similar to YS. Based on plumage colouration, we identified 37 birds as YS and 60 birds as BS, while 10 birds were classified as doubtful plumage birds.

### Cluster analysis

**Figure 4:**
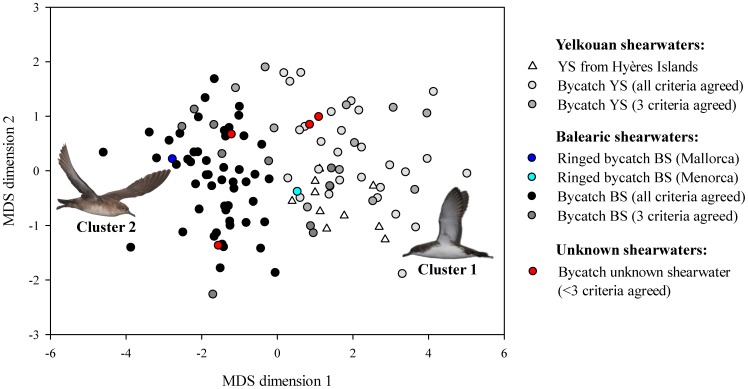
Multidimensional scaling similarity plot between Yelkouan (YS) and Balearic (BS) shearwaters based on biometric measurements. This plot was performed including all birds of dataset 1, i.e. 107 bycatch birds and 10 YS from Hyères Islands (n_total_ = 117). The colour of the symbols is based on mismatches of species identification across 4 different criteria (Table 2) with the exception of the bycatch birds ringed in Mallorca and Menorca (represented with dark blue and cyan circles, respectively) and the Yelkouan shearwaters breeding in Hyères islands (white triangles). The majority of the birds segregated according to their assigned species. Pictures courtesy of José Manuel Arcos (YS) and Verónica Cortés (BS).

The "pamk" function, used for estimating the optimal number of clusters, indicated the existence of two clusters in biometric, geometric morphometric, genetic and stable isotope data of birds from dataset 1. In the case of the linear biometric data, cluster 1 included the 10 YS breeding at Hyères Islands (see white triangles in Fig. 4), the bycatch BS ringed in Menorca (5070034, see cyan circle in Fig. 4) and 43 bycatch birds, while the cluster 2 included the bycatch BS ringed in Mallorca (5042990, see dark blue circle in Fig. 4) and 62 bycatch birds. The obtained clusters 1 and 2 showed Jaccard similarities of 0.970 and 0.973, respectively, indicating that they were highly stable.

**Figure 5:**
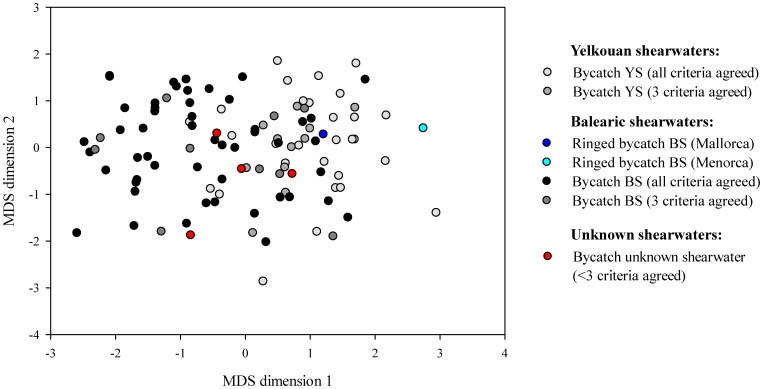
Multidimensional scaling similarity plot between Yelkouan (YS) and Balearic (BS) shearwaters based on geometric morphometric data. This plot was performed including all birds of dataset 1, i.e. 107 bycatch birds. The colour of the symbols depends of the mismatch of the species identification across 4 different criteria (Table 2) with the exception of the bycatch birds ringed in Mallorca and Menorca (represented with dark blue and cyan circles, respectively). Birds did not clearly segregated according to their assigned species.

Based on the geometric morphometrics data, cluster 1 included 54 bycatch birds, while the cluster 2 included the two BS ringed (see cyan and dark blue circle, in Fig. 5) and 51 bycatch birds. The bootstrap resampling scheme, however, indicated low stability of the clusters based on the Jaccard's similarity values (0.631 and 0.598 to cluster 1 and 2, respectively).

**Figure 6:**
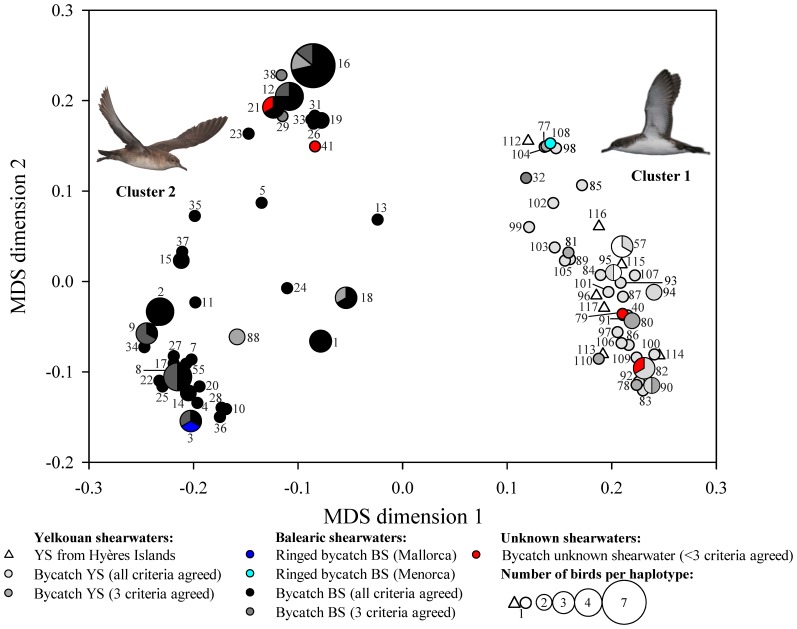
Multidimensional scaling similarity plot based on genetic data between Yelkouan (YS) and Balearic (BS) shearwaters of dataset 1. This plot includes all birds of dataset 1, i.e. 107 bycatch birds and 10 YS from Hyères Islands (n_total_ = 117) and has the same code colour of the symbols as in the Fig. 4. The size of the symbol represents the number of birds that shared the same haplotype (see S5 Table). The bycatch birds clearly segregated according to their assigned species and there is a significant match with birds of known species. Pictures courtesy of José Manuel Arcos (YS) and Verónica Cortés (BS).

**Figure 7:**
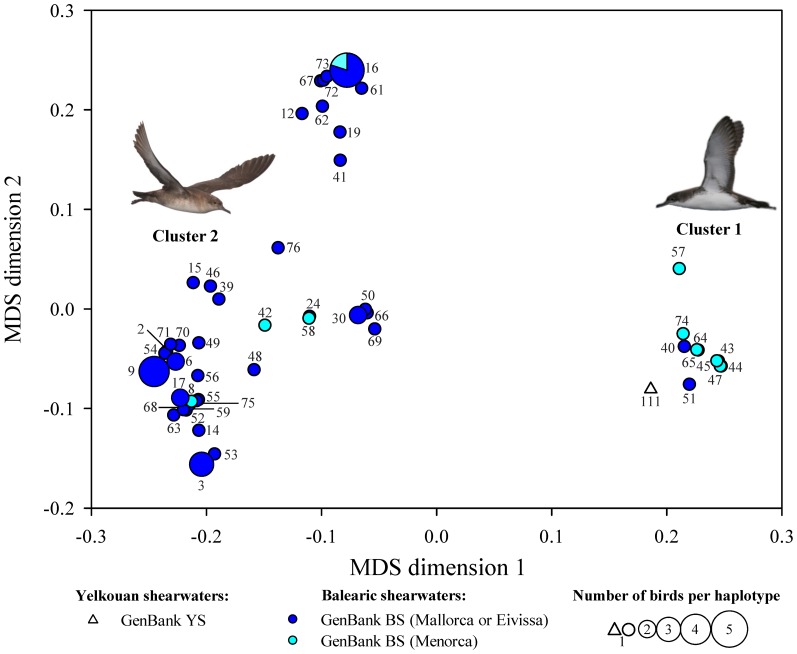
Multidimensional scaling similarity plot based on genetic data between Yelkouan (YS) and Balearic (BS) reference sequences from GenBank. This plot includes reference sequences from GenBank of 50 BS that were ringed in Mallorca, Eivissa or Formetera (dark blue) and 13 that were ringed in Menorca (cyan) and one YS from Hyères islands (white triangle). The size of the symbol represents the number of birds that shared the same haplotype (see S5 Table). The segregation of the reference sequences matched the one achieved with the bycatch birds (please see Fig. 6). Pictures courtesy of José Manuel Arcos (YS) and Verónica Cortés (BS).

Regarding to genetic data, the cluster 1 included all YS from Hyères Islands (see white triangles in Fig. 6 and 7), 39 bycatch birds, the bycatch BS ringed in Menorca (5070034, see cyan circle in Fig. 6), and nine and two BS obtained from GenBank that were ringed in Menorca and Mallorca, respectively (see Fig. 7 and S5 and S6 Tables). Cluster 2 included 66 bycatch birds, the bycatch BS ringed in Mallorca (5042990, see dark blue circle Fig. 6) and 48 reference sequence of BS obtained from GenBank that were ringed in Mallorca, Eivissa or Formentera and 4 that were ringed in Menorca respectively (see Fig. 7 and S5 and S6 Tables). The two clusters based on the mtDNA pairwise distance matrix showed Jaccard similarities of 1.000, indicating that they were highly stable.

**Figure 8:**
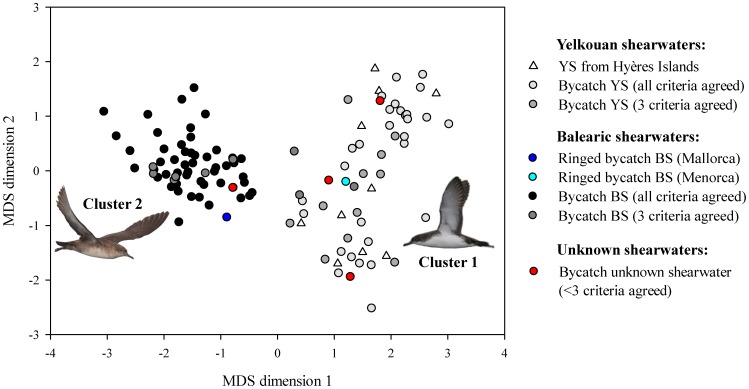
Multidimensional scaling similarity plot between Yelkouan (YS) and Balearic (BS) shearwaters based on stable isotopic data. This plot was performed including all birds of dataset 1, i.e. 107 bycatch birds and 10 YS from Hyères Islands (n_total_ = 117). The colour of the symbols is as in Fig. 4. Birds clearly segregated according to their assigned species. Pictures courtesy of José Manuel Arcos (YS) and Verónica Cortés (BS).

Concerning stable isotope clusters, the cluster 1 included the 10 YS from Hyères Islands (see white triangles in Fig. 8), the bycatch BS ringed in Menorca (5070034, see cyan circle in Fig. 8) and 46 bycatch birds, while the cluster 2 included the bycatch BS ringed in Mallorca (5042990, see dark blue circle in Fig. 8) and 59 bycatch birds. The clusters 1 and 2 showed Jaccard similarities of 0.982 and 0984, respectively, indicating that they were highly stable.

### Species identification

For species identification purpose of birds from dataset 1, we did not include the geometric morphometrics criterion, as the stability of the clusters obtained was low.

Linear biometry, stable isotope and genetic data assigned all birds of known YS species (Hyères Islands) to the cluster 1. Moreover 69.2% of the reference sequences from GenBank of BS ringed in Menorca were also assigned to this cluster. In contrast, 96.0% of the reference sequences of BS ringed in Mallorca, Eivissa or Formentera and the bycatch bird ringed in Mallorca were assigned to cluster 2. Therefore, we assumed that cluster 1 and 2 corresponded to YS and BS species, respectively.

Based on clusters results and plumage colouration, there was 82 out of 107 bycatch birds (76.6%) from dataset 1 that were consistently assigned to the same cluster by all criteria: 30 were assigned to YS and 52 to BS (see Table 2). Regarding to the remaining birds, 11 and 10 were assigned to YS and BS, respectively, by the agreement of three criteria (Table 2) and there were 4 birds for which it was not possible to infer their species as there were fewer than 3 criteria agreeing in the species identification (Table 2).

**Table 2:** Number of bycatch specimens showing the match and mismatch of the species identification across 4 different criteria.

Number and percentage of specimens	40* (34.2%)	52** (44.4%)	3 (2.6%)	4 (3.4%)	4 (3.4%)	1 (0.9%)	3 (2.6%)	4 (3.4%)	2 (1.7%)	1 (0.9%)	2 (1.7%)	1 (0.9%)
Plumage colouration	YS	BS	YS	YS	BS	BS	BS	?	?	?	?	?
Biometry	YS	BS	**BS**	YS	**YS**	BS	BS	YS	BS	**BS**	YS	BS
Genetic	YS	BS	YS	**BS**	BS	**YS**	BS	YS	BS	YS	**BS**	**YS**
Stable isotopes	YS	BS	YS	YS	BS	BS	**YS**	YS	BS	YS	YS	BS
Species assigned	YS	BS	YS	YS	BS	BS	BS	YS	BS	US	US	US

Number of bycatch specimens showing the match and mismatch of the species identification across 4 different criteria: plumage colouration, biometry, stable isotopes and genetics. YS – Yelkouan shearwater; BS – Balearic shearwater; US – unknown shearwater; “?” – doubtful plumage birds. Mismatches among criteria are marked in bold. The percentage of specimens is relative to the total number (117) of birds of dataset 1, i.e. 107 bycatch birds and 10 YS from Hyères Islands.* includes the 10 Yelkouan shearwaters from Hyères Islands and the bycatch Balearic shearwater ringed in Menorca. ** includes the bycatch Balearic shearwater ringed in Mallorca.

So, based on 113 birds that we were able to identify their species (including the YS from Hyères Is.), SIA was the criterion with more birds correctly identify (97%) followed by genetic analysis (96%), plumage colouration (95%) and biometry (94%).

### Differences between species

To check for differences between species, we used only birds of dataset 1 for which it was possible to infer their species (Table 2).

All linear biometric variables were significantly different between species and sexes (Table 3) with BS assigned individuals having greater biometric measures than YS assigned individuals and males being bigger than females (Table 2).

**Table 3:** Mean (± standard deviation) biometric measurements (mm) and their respective statistical results.

Species	Yelkouan shearwater		Balearic shearwater		MANOVA								
Sex	Males	Females	Males	Females	Species			Sex			Species*sex		
N	18	33	30	32	F	P	*η*_p_^2^	F	P	*η*_p_^2^	F	P	*η*_p_^2^
Bill depth at base (mm)	11.39±0.51	10.66±0.47	12.46±0.40	11.47±0.45	114.510	**<0.001**	0.512	94.090	**<0.001**	0.463	2.128	0.147	0.019
Bill depth at nostril (mm)	8.24±0.43	7.59±0.43	9.15±0.38	8.39±0.44	110.829	**<0.001**	0.504	74.940	**<0.001**	0.407	0.402	0.528	0.004
Bill length(mm)	38.01±1.13	36.63±1.38	39.57±1.34	37.47±1.22	23.138	**<0.001**	0.175	48.582	**<0.001**	0.308	2.129	0.147	0.019
Maximum head length (mm)	87.74±1.51	84.20±1.86	91.84±1.73	87.47±1.40	132.614	**<0.001**	0.549	152.830	**<0.001**	0.584	1.721	0.192	0.016
Tarsus length (mm)	48.52±1.52	47.07±1.17	50.65±1.37	49.24±1.24	72.373	**<0.001**	0.399	31.965	**<0.001**	0.227	0.007	0.934	<0.000
Wing length (mm)	241.3±5.6	237.5±4.7	252.3±4.3	251.9±5.1	181.797	**<0.001**	0.625	4.923	**0.029**	0.043	3.368	0.069	0.030

In this table we included all the Yelkouan shearwaters breeding in Hyères Islands (France), as well as all the bycatch birds that were equally assigned to the same species by at least three of criteria presented in table 2 (n_total_ = 113). All statistical significant results are marked in bold. Balearic shearwater showed significantly greater biometric measures that Yellkouan shearwaters and in both species males were significantly greater than females.

Concerning geometric morphometrics, both species and sexes showed significant differences in bill size with BS and males having a dorso-ventrally thicker bill than YS and female birds (see Table 4). For variables related to bill's shape, there was a significant difference between species (MANOVA: F = 5.677, p = <0.001 and *η*_p_^2^ = 0.571), but not between sexes (MANOVA: F = 1.173, p = 0.301 and *η*_p_^2^ = 0.216) nor interaction between species and sexes (MANOVA: F = 0.242, p = 0.999 and *η*_p_^2^ = 0.054).

**Table 4:** Mean (± standard deviation) centroid size (log) and its respective statistical results.

Species	Yelkouan shearwater		Balearic shearwater		Two-way Anova								
Sex	Male	Female	Male	Female	Species			Sex			Species*sex		
N	13	28	30	32	F	P	*η*_p_^2^	F	P	*η*_p_^2^	F	P	*η*_p_^2^
Centroid size	1.786±0.012	1.773±0.019	1.821±0.020	1.795±0.019	51.945	**<0.001**	0.344	24.647	**<0.001**	0.199	2.807	0.097	0.028

In this table we included only the bycatch birds that were equally assigned to the same species by at least three of criteria presented in table 2 (n_total_ = 103). For the 10 Yelkouan Shearwater breeding in Hyères Islands (France) we did not have geometric morphometric data and so they were not included in this table. All statistical significant results are marked in bold. Balearic shearwater showed significantly greater values of centroid size (i.e. bill size independent of bill shape) than Yellkouan shearwaters.

Carbon and nitrogen stable isotope values of feathers P1 and R6 differed significantly between species, with BS showing greater isotopic values than YS ones (p-values always <0.001, see Table 5).

**Table 5:** Mean (± standard deviation) carbon and nitrogen stable isotope values of the 1^st^ primary (P1) and the 6^th^ rectrix (R6).

Variable	Yelkouan shearwater		Balearic shearwater		Levene test		t-test		
	n	Mean	n	mean	F	P	t	D.f.	P
*δ* ^15^N_Air_ 1st primary (P1)	51	+11.3±0.9	62	+14.4±0.8	3.350	0.070	−18.838	111	**<0.001**
*δ* ^13^C_VPDB_ 1st primary (P1)	51	−18.2±1.2	62	−15.7±0.6	64.267	**<0.001**	−13.155	70.164	**<0.001**
*δ* ^15^N_Air_ 6th rectrix (R6)	51	+11.8±1.4	62	+14.1±1.1	5.549	**0.020**	−9.943	97.386	**<0.001**
*δ* ^13^C_VPDB_ 6th rectrix (R6)	51	−17.5±1.0	62	−16.0±0.6	17.368	**<0.001**	−9.565	82.534	**<0.001**

Mean (± standard deviation) carbon and nitrogen stable isotope values of the 1st primary (P1) and the 6th rectrix (R6) feathers of all the Yelkouan shearwaters breeding in Hyères Islands (France), as well as all the bycatch birds that were equally assigned to the same species by at least three of criteria presented in table 2 (n_total_ = 113). All statistical significant results are marked in bold, i.e., with p-value lower that 0.0125 (Bonferroni correction). Balearic shearwaters showed significantly more enriched carbon and nitrogen isotopic values in both feathers than Yelkouan shearwaters.

### Annual variation in stable isotope values

**Figure 9:**
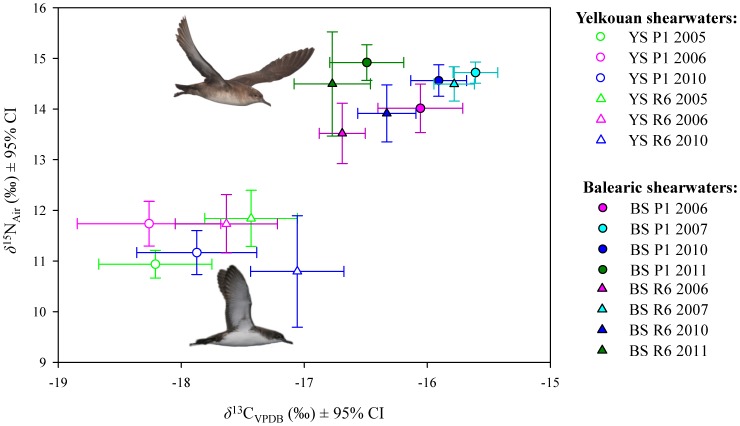
Mean (±95% CI) of *δ* ^15^N and *δ* ^13^C of P1 and R6 of Yelkouan (YS)and Balearic (BS)shearwaters from different years. In this figure we only represented the isotopic mean of years of moult that included at least 10 birds per year (to check the sample sizes, please see Table 1). Although that were differences between years, the differences between species were more important, and with the exception of *δ* ^13^C of R6, the isotopic values of the two species hardly overlapped. Pictures courtesy of José Manuel Arcos (YS) and Verónica Cortés (BS).

To increase the temporal series of birds with stable isotope data, we applied the DFAs obtained from birds of dataset 1 (for DFA results see Table S7 in Supporting Information) to the 77 additional shearwaters from years 2008 to 2011 (dataset 2). Out of these 77 birds, 65 were equally assigned to the same species by plumage colouration and by the DFAs. To understand whether annual variation in stable isotopes in feathers could influence species identification, we used all birds from both datasets (excluding only the unknown shearwaters, see Table 1) in the variance components estimation analysis to estimate the contribution of each random effect (species and year of moult) to the overall variability. The factor species always contributed more to the variance of the dependent variables than then the rest of factors, except for the nitrogen values of the R6 for which the error had the greater contribution (see S2 Fig.). This was also observed when plotting the isotopic results by species, feather and year of moult (only of years with sample sizes of at least 10 birds), where despite the within species annual variation observed in *δ* ^15^N and *δ* ^13^C values, the two species hardly overlapped (see Fig. 9).

## Discussion

In line with numerous previous studies, our study shows that species identification can be controversial and very sensitive to the applied method [72], [73]. It also highlights the need of integrative taxonomy as an essential part for the species identification process [26], [74]–[76], especially when examining recently diverged species. However, this study is among the first to show that stable isotope analyses can be used as a species identification tool and can provide evidence of recent ecological divergence in closely related species.

The most widely used and straightforward approach for bird identification is plumage colouration [77], which has been traditionally used to identify birds. In our sample species identification based on plumage colouration was in most cases limited by the wet and bloody plumage of the birds bycaught in longliners, which precluded a quantitative evaluation of plumage coloration. Still, we were able to assign 97 out of 107 birds (90.7%) to one of the species using plumage colouration. When closely related species differ through a diffuse gradient of traits that overlap to some extent, the identification may be difficult, subjective or even impossible. Moreover, species identification using plumage colouration can be relatively subjective, depending on the experience of the observer, the wear of the feathers during moulting and even light conditions. This is the case of the YS and the whitish BS, where the identification by plumage colouration has proven difficult, especially during the spring [35].

When birds can be examined in-hand, biometric measurements can be easily used to tease apart species differing in their morphology (e.g. [78]). In the case of the studied species, in general, BS showed significantly larger biometric measures than YS ones and simple measurements on the tarsus, the wing and the bill segregated the two species in two different clusters (see Fig. 4 and Table 3). By using the discriminant function analysis we were able to discriminate between birds assigned to YS or BS based on two measurements: the wing length and the bill depth at nares. Although it was already known that these two species showed different biometric measurements [34], [37], [38], [79], no discriminant function was available until now. However this discriminant function should be used with caution as BS shows a high correlation between body size and breeding colony [34], with birds from Menorca showing similar biometry with the ones from neighbouring colonies of YS [34]. Indeed, in our data, the only bycatch bird that was ringed in Menorca was assigned as YS. Moreover, this discriminant function analysis may also be affected in the case of existing a bias associated to the body size of birds that feed on baits of longliners.

Geometric morphometrics is a novel approach to quantify not only variation in shape, but also differences in size. This method was already used to detect subtle sexual dimorphism in the bill of another shearwater species, the Cory's shearwater (*Calonectris diomedea borealis*, [80]). In our study, the geometric morphometric data indicated the presence of two clusters, however their stability was very low and we discarded this criterion for subsequent analyses. Using birds of unknown origin probably increased geographic variation in bill shape and size limiting the power of this approach. For example, BS show high correlation between biometry and breeding colony [34], if the same happens with bill shape, this can reduce the identification power of geometric morphometrics. Furthermore, the need to use landmarks only in rigid structures, such as the bill, and the ecological conditions experienced by different colonies may also limit the identification power of geometric morphometrics, as bill shape is known to be ecologically plastic [81].

Genetic approaches have become essential tools to resolve taxonomic uncertainties and define strategies for species conservation management [17], [82]. In our study, the genetic analyses, based on a small fragment of the hypervariable control region of the mitochondrial DNA, resulted in a clear separation between YS and BS that mostly agrees with species identification based on other methods and that was confirmed by the inclusion of reference sequences of the two shearwaters of known origin in the analysis. The majority of the reference birds were clustered according to their species, all the birds of known YS species (from Hyères) clustered all together and in a different cluster of the 96.0% of the BS ringed in Mallorca, Eivissa or Formentera. But 69.2% of BS from Menorca clustered with birds of known YS species, which also occurs in other studies of these species [34]. This misclassification rate is also concordant with the low levels of divergence observed between these two species, when compared to other well-recognized seabird species [83], as well as with the reported frequent hybridization between them on Menorca [34], [38]. This genetic pattern is further supported by ecological and phenotypic evidence such as the occurrence of interspecific breeding pairs [34], [38] and intermediate phenotypes [38]. Put together, these findings support the idea that hybridization may be a common phenomenon between these two recently diverged species, which highlight the difficulty in identify these two species and thus the potential implications for conservation.

Stable isotopes can be useful intrinsic markers of the spatial and trophic ecology when species feed on isotopically different preys or in areas with distinct baseline isotopic values [21]. Baseline isotopic values usually differ among oceanic areas [84] and determine the isotopic forms integrated in prey, which in turn are readily deposited into the growing feathers of the birds in a predictable manner. In the present study, we analyzed *δ* ^13^C and *δ* ^15^N of two feathers, one inferred to be grown at the beginning and the other normally at the end of the non-breeding season [42]. After the breeding period, the majority of YS and BS migrate to different oceanic areas, encompassing the Black and Aegean Seas and the Iberian Peninsula and Bay of Biscay, respectively [39]–[42]. Since these areas show different basal isotopic values [42], we would expect interspecific differences in the isotopic values of their feathers. Accordingly, this study showed that the isotopic combination of the two feathers analysed allowed the separation of YS and BS into two stable clusters. This is further supported when contrasting these clusters with the reference specimens of known species, which in general grouped with their corresponding species cluster, with the exception of the bycatch bird ringed in Menorca. Regarding to the discriminant functions obtained from SIA data, although the percentage of correct identification was high, they should be considered with caution, as some BS from Menorca or other colonies may have similar isotopic values to the YS resulting from few individuals from both species that may remain year-round in western Mediterranean waters [40], [42]. Moreover, BS spend the non-breeding period in at least two different areas in the NE Atlantic Ocean [39] and it is still unclear whether these areas show different baseline isotopic values. If one of those areas shows similar isotopic values to the ones of W Mediterranean, the differentiation between the two species would be more difficult. Further studies are needed to examine stable isotope of feathers of YS and BS from different colonies tracked with geolocators to improve the discriminant function here presented.

We contend that SIA can be a complementary and powerful tool for species identification in a number of animal species worldwide [22], [23], [85]. This approach, however, has drawbacks to species identification, such as the variability in isotopic values over the years and the lack of knowledge or differences in isotopic values among different food resources or among foraging areas. In this study, we went deeper in examining the stability in the isotopic values of the feathers through time. Our results indicated annual variability in stable isotope values, but differences over time were smaller than differences between species (Fig. 9 and S2 Fig.). These variability between years may result from temporal variation in baseline isotopic values in the non-breeding areas. Alternatively, differences among years may arise from annual differences among individuals in the use of non-breeding areas with distinct baseline levels or prey availability. While we are confident that temporal variability had little impact on the assignment of birds to our study species, the utility of this approach can be limited in cases where the isotopic segregation between species is less pronounced and variability in stable isotope values among years is high. Therefore we strongly recommend to check temporal trends in stable isotopic values, if possible within individuals, as a preliminary step before applying this approach for species identification.

The congruence between at least three criteria in species identification allowed to infer the species of the majority of the bycatch birds (96.3% and 84.4% of birds from dataset 1 and 2, respectively). However there was some incongruities among criteria which may result from the overlap of both species in some traits [34] or to the known hybridization between the two species in Menorca [38]. Indeed, the bycatch bird ringed in Menorca was assigned as YS by plumage colouration, biometry, genetic and isotopic criteria and clustered with (1) birds of known YS species and; (2) the majority of reference sequences of BS from Menorca. This is in line with other studies suggesting that at least part of the population breeding in Menorca actually belong to YS species [86].

In conclusion, our results indicate the importance of using integrative taxonomy in species identification, especially of recently diverged species. The use of several criteria can help to overcome the drawbacks of each approach alone, as each criterion will measure different biological phenomena. This is very useful, as species divergence does not occur simultaneously in genetic, ecological or morphological traits [87]. Our findings also have relevant conservation implications for risk management and planning since the ability to identify birds to the right species is essential for the careful evaluation of anthropogenic impacts, such as human activities, pollution or the introduction of exotic species.

## Supporting Information

S1 Fig **Photography and description of biometric measurements.**
(DOCX)

S2 Fig **Estimate variance (%) of** ***δ*** **^15^N and** ***δ*** **^13^C of P1 and R6 regarding to random factors: species and year of moult.**
(DOCX)

S1 Table **Biometric measurements of each bycatch shearwaters and Yelkouan shearwaters from Hyères islands (France).**
(XLSX)

S2 Table **Intra- and inter-observer errors and paired t-test of the biometric measurements.**
(DOCX)

S3 Table **Description of the landmarks and semilandmarks used to delineate the bill shape.**
(DOCX)

S4 Table **Accepted and mean measured values of the standard material for SIA.**
(DOCX)

S5 Table **Description of the number of genetic sequences for each haplotype.**
(DOCX)

S6 Table **Ringing information of the ringed shearwaters and of reference sequences of GenBank.**
(XLSX)

S7 Table **Discriminant function analysis results and respective Fisher's classification function coefficients.**
(DOCX)
