# Supplementary material for: Comparing Multiple Criteria for Species Identification in Two Recently Diverged Seabirds
Source: PLoS One. 2014 Dec 26;9(12):e115650. doi: 10.1371/journal.pone.0115650 (PMC4277347; doi:10.1371/journal.pone.0115650)

**Comparing multiple criteria for species identification in two recently diverged seabirds**

Teresa Militão, Elena Gómez-Díaz, Antigoni Kaliontzopoulou, Jacob González-Solís

Supporting information:

**Figure S1. Photography and description of biometric measurements.** Measurements of tarsus length (A - from the depression in the angle of the intertarsal joint to the base of the last complete scale before the toes diverge), head-bill length (B - length from the cerebellum roof to the tangent of the outermost anterior hook), bill length (C - from the tip of the hook to the edge of the first feather implantation), wing length (D - maximum flattened and straightened chord from carpal joint to the tip of the longest primary), bill depth at nostril (E - bill depth from the lower anterior border of the nostril) and bill depth at base (F - maximum bill depth at the upper posterior border of the nostril in the edge of the first feather implantation).


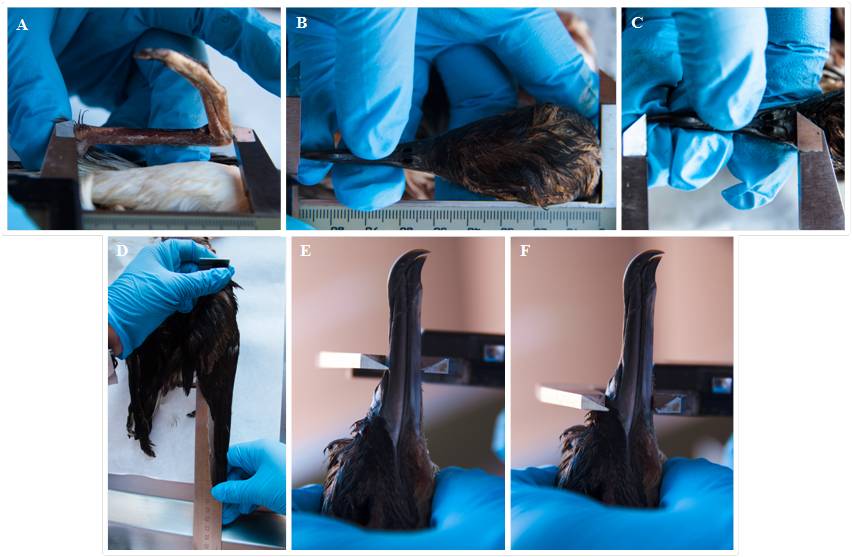

Supplement: S1 Fig — Photography and description of biometric measurements. (DOCX) [file pone.0115650.s001.docx]
