# Supplementary material for: Comparing Multiple Criteria for Species Identification in Two Recently Diverged Seabirds
Source: PLoS One. 2014 Dec 26;9(12):e115650. doi: 10.1371/journal.pone.0115650 (PMC4277347; doi:10.1371/journal.pone.0115650)
Supplement: S2 Fig — Estimate variance (%) of δ 15N and δ 13C of P1 and R6 regarding to random factors: species and year of moult. (DOCX) [file pone.0115650.s002.docx]

**Comparing multiple criteria for species identification in two recently diverged seabirds**

Teresa Militão, Elena Gómez-Díaz, Antigoni Kaliontzopoulou, Jacob González-Solís

Supporting information:

**Figure S2. Estimate variance (%) of *δ*^15^N and *δ*^13^C of P1 and R6 regarding to the random factors: species and year of moult.** Although the factor year of moult contributed to some variance of the isotopic values of the first primary feathers (P1) and sixth rectrix (R6) the factor species was always the most important, with the exception of *δ*^13^C of the R6, in which error was most important factor to the variance of this variable.
