# Supplementary material for: Comparing Multiple Criteria for Species Identification in Two Recently Diverged Seabirds
Source: PLoS One. 2014 Dec 26;9(12):e115650. doi: 10.1371/journal.pone.0115650 (PMC4277347; doi:10.1371/journal.pone.0115650)
Supplement: S2 Table — Intra- and inter-observer errors and paired t-test of the biometric measurements. (DOCX) [file pone.0115650.s004.docx]

**Comparing multiple criteria for species identification in two recently diverged seabirds**

Teresa Militão, Elena Gómez-Díaz, Antigoni Kaliontzopoulou, Jacob González-Solís

**Table S2- Results of the intraclass correlation and paired t-test of the intra- (A) and inter-observer (B) error of the biometric measurements.** All the measurements significantly different are marked in bold, i.e., with p-value lower that 0.008 (Bonferroni correction). All the measurements showed high intraclass correlation (>0.9 from 0 to 1), but some measurements showed different differences within and between observers.

| A | Observer 1 (TM) | | | | | | | |
| --- | --- | --- | --- | --- | --- | --- | --- | --- |
|  | Measurement 1 | | | | | | | |
| Measurement 2 | n | Statistic test | Bill depth at base | Bill depth at nostril | Bill length | Maximum head length | Tarsus length | Wing length |
|  | 85 | ICC | 0.952 | 0.975 | 0.985 | 0.996 | 0.980 | 0.988 |
|  | 85 | Paired t-test | t_84_=9.040 **P<0.001** | t_84_=3.484 **P=0.001** | t_84_=4.767 **P<0.001** | t_84_=5.964 **P<0.001** | t_84_=6.000 **P<0.001** | t_84_=-12.247 **P<0.001** |
|  | Observer 2 (JGS) | | | | | | | |
|  | Measurement 1 | | | | | | | |
| Measurement 2 | n | Statistic test | Bill depth at base | Bill depth at nostril | Bill length | Maximum head length | Tarsus length | Wing length |
|  | 37 | ICC | 0.914 | 0.960 | 0.943 | 0.972 | 0.959 | 0.984 |
|  | 37 | Paired t-test | t_36_=0.120 P=0.906 | t_36_=2.093 P=0.043 | t_36_=0.766 P=0.449 | t_36_=-2.022 P=0.051 | t_36_=-5.285 **P<0.001** | t_36_=1.598 P=0.119 |

| B | Observer 1 (TM) | | | | | | | |
| --- | --- | --- | --- | --- | --- | --- | --- | --- |
|  | Measurement 1 | | | | | | | |
| Observer 2 (JGS) | n | Statistic test | Bill depth at base | Bill depth at nostril | Bill length | Maximum head length | Tarsus length | Wing length |
| Measurement 1 | 85 | ICC | 0.921 | 0.959 | 0.959 | 0.996 | 0.963 | 0.988 |
|  | 85 | Paired t-test | t_84_=-1.556 P=0.124 | t_84_=2.205 P=0.030 | t_84_=-2.978 **P=0.004** | t_84_=16.980 **P<0.001** | t_84_=5.820 **P<0.001** | t_84_=0.623 P=0.535 |
| Measurement 2 | 37 | ICC | 0.936 | 0.938 | 0.966 | 0.971 | 0.984 | 0.99 |
|  | 37 | Paired t-test | t_36_=0.347 P=0.731 | t_36_=3.346 **P=0.002** | t_36_=-0.620 P=0.539 | t_36_=2.804 **P=0.008** | t_36_=3.740 **P=0.001** | t_36_=1.373 P=0.178 |
|  | Measurement 2 | | | | | | | |
| Observer 2 (JGS) | n | Statistic test | Bill depth at base | Bill depth at nostril | Bill length | Maximum head length | Tarsus length | Wing length |
| Measurement 1 | 85 | ICC | 0.927 | 0.972 | 0.966 | 0.998 | 0.968 | 0.982 |
|  | 85 | Paired t-test | t_84_=-7.234 **P<0.001** | t_84_=-0.700 P=0.486 | t_84_=-6.231 **P<0.001** | t_84_=16.348 **P<0.001** | t_84_=2.065 P=0.042 | t_84_=8.181 **P<0.001** |
| Measurement 2 | 37 | ICC | 0.913 | 0.964 | 0.956 | 0.976 | 0.983 | 0.980 |
|  | 37 | Paired t-test | t_36_=-4.506 **P<0.001** | t_36_=2.078 P=0.045 | t_36_=-3.387 **P=0.002** | t_36_=1.076 P=0.289 | t_36_=-0.891 P=0.379 | t_36_=7.474 **P<0.001** |
