# Supplementary material for: Comparing Multiple Criteria for Species Identification in Two Recently Diverged Seabirds
Source: PLoS One. 2014 Dec 26;9(12):e115650. doi: 10.1371/journal.pone.0115650 (PMC4277347; doi:10.1371/journal.pone.0115650)
Supplement: S3 Table — Description of the landmarks and semilandmarks used to delineate the bill shape. (DOCX) [file pone.0115650.s005.docx]

**Comparing multiple criteria for species identification in two recently diverged seabirds**

Teresa Militão, Elena Gómez-Díaz, Antigoni Kaliontzopoulou, Jacob González-Solís

Table S3 – Description of the landmarks and semilandmarks used to delineate the bill shape for the geometric morphometric approach (see Fig. 3).

| Landmark no. | Description |
| --- | --- |
| 1 | Distal tip of superior unguicorn (maxillary unguis). |
| 2 | Distal tip of inferior unguicorn (mandibular unguis). |
| 3 | Anterior tip of inferior ramicorn in the juncture with inferior unguicorn. |
| 4 | Posterior tip of inferior ramicorn. |
| 5 | Posterior tip of latericorn. |
| 6 | Point of joint between the lower posterior border of nare with the superior and posterior point of the latericorn |
| 7 | Upper posterior border of nares. |
| 8 | Lower anterior border, in the joint with latericorn. |
| 9 | Joint between the anterior tip of latericorn and the superior and inferior unguicorn |
| 10 | Joint between upper anterior culminicorn and superior unguicorn. |
| 11, 12 and 13 | Equidistant semilandmarks on the curvature of the maxillary unguis, sliding between landmarks 1 and 10. |
