# Supplementary material for: Comparing Multiple Criteria for Species Identification in Two Recently Diverged Seabirds
Source: PLoS One. 2014 Dec 26;9(12):e115650. doi: 10.1371/journal.pone.0115650 (PMC4277347; doi:10.1371/journal.pone.0115650)
Supplement: S4 Table — Accepted and mean measured values of the standard material for SIA. (DOCX) [file pone.0115650.s006.docx]

**Comparing multiple criteria for species identification in two recently diverged seabirds**

Teresa Militão, Elena Gómez-Díaz, Antigoni Kaliontzopoulou, Jacob González-Solís

**Table S4 - Accepted and measured values of the standard material used in the stable isotopic analysis performed in this study.** Accepted and mean measured (±standard deviation) values of the standard material used in the stable isotopic analysis performed in this study, as well as the mean minimum and maximum values obtain in each run. The "n" refers to the number of samples of standards materials used.

| Standard material name | *δ*^15^N_Air_ (‰) | | | | *δ*^13^C_VPDB_ (‰) | | | | References |
| --- | --- | --- | --- | --- | --- | --- | --- | --- | --- |
|  | Accepted value ± SD | Measured values | | | Accepted value ± SD | Mean measured values | | |  |
|  |  | n | Mean of all runs ± SD | Minimum - maximum mean within runs |  | n | Mean of all runs ± SD | Minimum - maximum mean within runs |  |
| IAEA CH6 |  |  |  |  | –10.449±0.033 | 22 | –10.4±0.2 | –10.6 to –10.3 | [1] |
| IAEA CH7 |  |  |  |  | –32.151±0.050 | 24 | –32.0±0.2 | –32.2 to –31.7 | [1] |
| USGS 40 | –4.52±0.06 | 17 | –4.5±0.2 | –4.7 to -4.2 | –26.24±0.07 | 17 | –26.1±0.2 | –26.3 to –25.9 | [2] |
| IAEA N1 | +0.43±0.07 | 20 | +0.4±0.1 | 0.2 to 0.5 |  |  |  |  | [3] |
| IAEA N2 | +20.41±0.12 | 23 | +19.9±0.3 | 19.4 to 20.2 |  |  |  |  | [3] |
| IAEA NO3 | +4.72±0.13 | 17 | +4.5±0.2 | 4.0 to 4.7 |  |  |  |  | [3] |

Reference List

1. Coplen TB, Brand WA, Gehre M, Gröning M, Meijer HAJ, Toman B, Verkouteren RM (2006) New guidelines for ^13^C measurements. Anal Chem 78: 2439-2441.

2. Qi H, Coplen TB, Gelimann H, Brand WA, Böhlke JK (2003) Two new organic reference materials for δ^13^C and δ^15^N measurements and new value for the δ^13^C of NBS 22 oil. Rapid Commun Mass Spectrom 17: 2483-2487.

3. Böhlke JK, Coplen TB (1993) Interlaboratory comparison of reference materials for nitrogen-isotope-ratio measurements. Reference and intercomparison materials for stable isotopes of light elements Proceedings of a consultants meeting Viena, Austria. pp.51-66.
