# Supplementary material for: Comparing Multiple Criteria for Species Identification in Two Recently Diverged Seabirds
Source: PLoS One. 2014 Dec 26;9(12):e115650. doi: 10.1371/journal.pone.0115650 (PMC4277347; doi:10.1371/journal.pone.0115650)
Supplement: S5 Table — Description of the number of genetic sequences for each haplotype. (DOCX) [file pone.0115650.s007.docx]

**Comparing multiple criteria for species identification in two recently diverged seabirds**

Teresa Militão, Elena Gómez-Díaz, Antigoni Kaliontzopoulou, Jacob González-Solís

**Table S5 – Description of the number of genetic sequences of birds bycatch, from Hyères Islands and those obtained from GenBank for each haplotype (see Fig. 6 and 7).** The birds bycatch were classified based on the mismatch table of the four criteria used for species assignation (see Table 2; YS – Yelkouan Shearwater; BS – Balearic Shearwater), the birds for which there was no agreement between criteria (< than 3 criteria agreeing) were considered unknown shearwaters.

| ID Haplotype | Bycatch birds | | | | | Birds of known species | | |
| --- | --- | --- | --- | --- | --- | --- | --- | --- |
|  | YS | | BS | | Unknown shearwaters | YS | BS GenBank Accession No. | |
|  | All criteria agreed | Three criteria agreed | All criteria agreed | Three criteria agreed | < three criteria agreed | Ringed at Hyères Islands | Ringed at Mallorca, Eivissa or Formentera | Ringed at Menorca |
| 1 |  |  | 3 |  |  |  |  |  |
| 2 |  |  | 4 |  |  |  | DQ230148 |  |
| 3 |  |  | 2 (including the one ringed in Mallorca) | 1 |  |  | DQ230132, DQ230137, DQ230171 |  |
| 4 |  |  | 1 |  |  |  |  |  |
| 5 |  |  | 1 |  |  |  |  |  |
| 6 |  |  |  |  |  |  | DQ230161, DQ230172 |  |
| 7 |  |  | 1 |  |  |  |  |  |
| 8 |  |  | 2 | 2 |  |  |  | DQ230182 |
| 9 |  |  | 1 | 2 |  |  | DQ230154, DQ230160, DQ230163, DQ230185 |  |
| 10 |  |  | 1 |  |  |  |  |  |
| 11 |  |  | 1 |  |  |  |  |  |
| 12 |  |  | 3 | 1 |  |  | DQ230156 |  |
| 13 |  |  | 1 |  |  |  |  |  |
| 14 |  |  | 2 |  |  |  | DQ230155 |  |
| 15 |  |  | 2 |  |  |  | DQ230150 |  |
| 16 |  | 1 | 5 | 1 |  |  | DQ230136, DQ230138, DQ230157, DQ230173, | DQ230178 |
| 17 |  |  | 1 |  |  |  | DQ230175, DQ230176 |  |
| 18 |  | 1 | 2 |  |  |  |  |  |
| 19 |  |  | 2 |  |  |  | DQ230174 |  |
| 20 |  |  | 1 |  |  |  |  |  |
| 21 |  |  | 2 |  | 1 |  |  |  |
| 22 |  |  | 1 |  |  |  |  |  |
| 23 |  |  | 1 |  |  |  |  |  |
| 24 |  |  | 1 |  |  |  | DQ230162 |  |
| 25 |  |  | 1 |  |  |  |  |  |
| 26 |  |  | 1 |  |  |  |  |  |
| 27 |  |  | 1 |  |  |  |  |  |
| 28 |  |  | 1 |  |  |  |  |  |
| 29 |  |  |  | 1 |  |  |  |  |
| 30 |  |  |  |  |  |  | DQ230131, DQ230168 |  |
| 31 |  |  | 1 |  |  |  |  |  |
| 32 |  |  |  | 1 |  |  |  |  |
| 33 |  |  | 1 |  |  |  |  |  |
| 34 |  |  | 1 |  |  |  |  |  |
| 35 |  |  | 1 |  |  |  |  |  |
| 36 |  |  | 1 |  |  |  |  |  |
| 37 |  |  | 1 |  |  |  |  |  |
| 38 |  |  |  | 1 |  |  |  |  |
| 39 |  |  |  |  |  |  | DQ230133 |  |
| 40 | 1 |  |  |  |  |  | DQ230134 |  |
| 41 |  |  |  |  | 1 |  | DQ230135 |  |
| 42 |  |  |  |  |  |  |  | DQ230139 |
| 43 |  |  |  |  |  |  |  | DQ230140 |
| 44 |  |  |  |  |  |  |  | DQ230141 |
| 45 |  |  |  |  |  |  |  | DQ230142 |
| 46 |  |  |  |  |  |  | DQ230145 |  |
| 47 |  |  |  |  |  |  |  | DQ230146 |
| 48 |  |  |  |  |  |  | DQ230147 |  |
| 49 |  |  |  |  |  |  | DQ230149 |  |
| 50 |  |  |  |  |  |  | DQ230151 |  |
| 51 |  |  |  |  |  |  | DQ230152 |  |
| 52 |  |  |  |  |  |  | DQ230153 |  |
| 53 |  |  |  |  |  |  | DQ230159 |  |
| 54 |  |  |  |  |  |  | DQ230164 |  |
| 55 |  |  | 1 |  |  |  | DQ230169 |  |
| 56 |  |  |  |  |  |  | DQ230177 |  |
| 57 | 1 |  |  |  |  | 2 |  | DQ230179 |
| 58 |  |  |  |  |  |  |  | DQ230180 |
| 59 |  |  |  |  |  |  | DQ230181 |  |
| 60 |  |  |  |  |  |  |  | DQ230184 |
| 61 |  |  |  |  |  |  | DQ230186 |  |
| 62 |  |  |  |  |  |  | DQ230199 |  |
| 63 |  |  |  |  |  |  | DQ230200 |  |
| 64 |  |  |  |  |  |  |  | DQ230201 |
| 65 |  |  |  |  |  |  |  | DQ230202 |
| 66 |  |  |  |  |  |  | DQ230203 |  |
| 67 |  |  |  |  |  |  | DQ230204 |  |
| 68 |  |  |  |  |  |  | DQ230205 |  |
| 69 |  |  |  |  |  |  | DQ230206 |  |
| 70 |  |  |  |  |  |  | DQ230207 |  |
| 71 |  |  |  |  |  |  | DQ230209 |  |
| 72 |  |  |  |  |  |  | DQ230210 |  |
| 73 |  |  |  |  |  |  | DQ230211 |  |
| 74 |  |  |  |  |  |  |  | DQ230214 |
| 75 |  |  |  |  |  |  | DQ230215 |  |
| 76 |  |  |  |  |  |  | DQ230217 |  |
| 77 |  | 1 |  |  |  |  |  |  |
| 78 |  | 1 |  |  |  |  |  |  |
| 79 |  |  |  |  | 1 |  |  |  |
| 80 |  | 2 |  |  |  |  |  |  |
| 81 |  | 1 |  |  |  |  |  |  |
| 82 | 2 |  |  |  | 1 |  |  |  |
| 83 | 1 |  |  |  |  |  |  |  |
| 84 | 1 |  |  |  |  |  |  |  |
| 85 | 1 |  |  |  |  |  |  |  |
| 86 | 1 |  |  |  |  |  |  |  |
| 87 | 1 |  |  |  |  |  |  |  |
| 88 |  | 2 |  |  |  |  |  |  |
| 89 | 1 |  |  |  |  |  |  |  |
| 90 | 1 | 1 |  |  |  |  |  |  |
| 91 | 1 |  |  |  |  |  |  |  |
| 92 | 1 |  |  |  |  |  |  |  |
| 93 | 1 |  |  |  |  |  |  |  |
| 94 | 2 |  |  |  |  |  |  |  |
| 95 | 1 |  |  |  |  | 1 |  |  |
| 96 |  |  |  |  |  | 1 |  |  |
| 97 | 1 |  |  |  |  |  |  |  |
| 98 | 1 |  |  |  |  |  |  |  |
| 99 | 1 |  |  |  |  |  |  |  |
| 100 | 1 |  |  |  |  |  |  |  |
| 101 | 1 |  |  |  |  |  |  |  |
| 102 | 1 |  |  |  |  |  |  |  |
| 103 | 1 |  |  |  |  |  |  |  |
| 104 | 1 |  |  |  |  |  |  |  |
| 105 | 1 |  |  |  |  |  |  |  |
| 106 | 1 |  |  |  |  |  |  |  |
| 107 | 1 |  |  |  |  |  |  |  |
| 108 | 1 (BS ringed in Menorca) |  |  |  |  |  |  |  |
| 109 | 1 |  |  |  |  |  |  |  |
| 110 |  | 1 |  |  |  |  |  |  |
| 111 |  |  |  |  |  | 1 |  |  |
| 112 |  |  |  |  |  | 1 |  |  |
| 113 |  |  |  |  |  | 1 |  |  |
| 114 |  |  |  |  |  | 1 |  |  |
| 115 |  |  |  |  |  | 1 |  |  |
| 116 |  |  |  |  |  | 1 |  |  |
| 117 |  |  |  |  |  | 1 |  |  |
