# Supplementary material for: Comparing Multiple Criteria for Species Identification in Two Recently Diverged Seabirds
Source: PLoS One. 2014 Dec 26;9(12):e115650. doi: 10.1371/journal.pone.0115650 (PMC4277347; doi:10.1371/journal.pone.0115650)
Supplement: S7 Table — Discriminant function analysis results and respective Fisher's classification function coefficients. (DOCX) [file pone.0115650.s009.docx]

**Comparing multiple criteria for species identification in two recently diverged seabirds**

Teresa Militão, Elena Gómez-Díaz, Antigoni Kaliontzopoulou, Jacob González-Solís

**Table S7 - Discriminant function analysis results and respective Fisher's classification function coefficients.** The discriminant function analysis (DFA) were constructed based on linear biometric measurements and stable isotope data of birds of dataset 1 that were assigned to the same specie by at least three criteria (see Table 2). Both DFA showed high values of correct species identification in the training and test data.

| Criterion | Species | Training data (cross-validation) | | Test data | | Fisher's classification function coefficients |
| --- | --- | --- | --- | --- | --- | --- |
|  |  | n | Correct identification (%) | n | Correct identification (%) |  |
| Biometry | YS | 40 | 91.1 | 11 | 100.0 | D = 9.578*Bill depth at base+8.582*Wing length-1078.186 |
|  | BS | 50 |  | 12 |  | D = 11.272*Bill depth at base+9.004*Wing length-1200.957 |
| Stable isotope | YS | 40 | 96.7 | 11 | 100.0 | D = 12.650**δ*^15^N (P1) - 21.198**δ*^13^C (P1) - 0.089**δ*^15^N (R6) - 265.992 |
|  | BS | 50 |  | 12 |  | D = 15.506**δ*^15^N (P1) - 17.724**δ*^13^C (P1) + 1.602**δ*^15^N (R6) - 263.371 |
